# An Introduction to Probabilistic Record Linkage with a Focus on Linkage Processing for WTC Registries

**DOI:** 10.3390/ijerph17186937

**Published:** 2020-09-22

**Authors:** Jana Asher, Dean Resnick, Jennifer Brite, Robert Brackbill, James Cone

**Affiliations:** 1 Department of Mathematics and Statistics, Slippery Rock University, Slippery Rock, PA 16057, USA; 2 National Opinion Research Center, Boston, MA 02114, USA; resnick-dean@norc.org; 3 Division of Epidemiology, New York City Department of Health and Mental Hygiene, World Trade Center Health Registry, New York, NY 11101, USA; jbrite@health.nyc.gov (J.B.); rbrackbi@health.nyc.gov (R.B.); jcone@health.nyc.gov (J.C.)

**Keywords:** epidemiology, disaster epidemiology, data matching, record linkage, probabilistic record linkage, interagency cooperation, 9/11 health

## Abstract

Since its post-World War II inception, the science of record linkage has grown exponentially and is used across industrial, governmental, and academic agencies. The academic fields that rely on record linkage are diverse, ranging from history to public health to demography. In this paper, we introduce the different types of data linkage and give a historical context to their development. We then introduce the three types of underlying models for probabilistic record linkage: Fellegi-Sunter-based methods, machine learning methods, and Bayesian methods. Practical considerations, such as data standardization and privacy concerns, are then discussed. Finally, recommendations are given for organizations developing or maintaining record linkage programs, with an emphasis on organizations measuring long-term complications of disasters, such as 9/11.

## 1. Introduction

**Figure 1:**
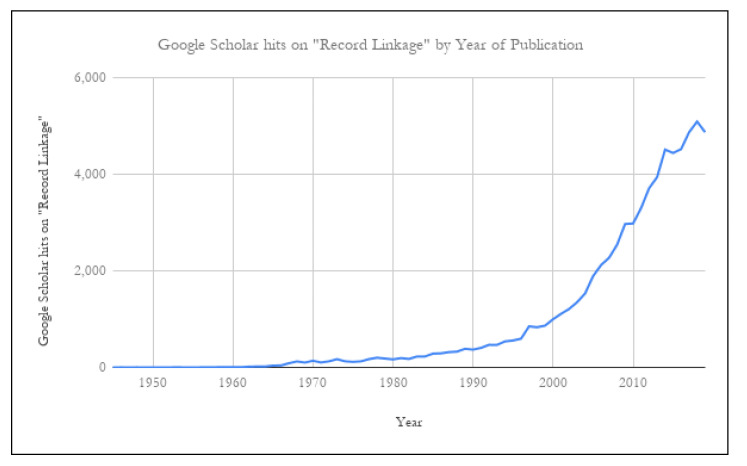
Publications related to record linkage on Google Scholar, by year of publication. Data obtained 9 August 2019 for years prior to 2019; data obtained 31 December 2019 for 2019.

From its humble beginnings in post-World War II public health research, the field of “record linkage”—that is, the matching of records for unique entities (typically people, but sometimes organizations, addresses, or something else)across one or more lists—has exploded into a multi-field research focus (see Figure 1). As the need for this technique has grown, fueled by ever improving computational capacity, more and more individual disciplines have begun their own foray into this process, including many researchers in the World Trade Center (WTC) community, defined as the researchers studying the ongoing effects on physical and mental health of exposure to the initial and later environment surrounding the 9/11 terrorist attacks.

Among the major 9/11 health cohorts, linkages are currently underway or have been conducted to cancer registries, the National Death Index, New York State hospitalizations, and various vital records. Moreover, several joint studies are being formulated to study pooled patient populations across cohorts. The reasons for this are both scientific and practical. As more data become available electronically and computational power improves, access to health data, at least from a technical point of view, has become easier. This is fortuitous as maintaining a large-scale research project over multiple decades among a trauma-exposed and aging population presents several challenges, chief among them attrition and reporting bias due to failing memories among respondents.

This paper introduces the field of records linkage to the reader, assuming little previous experience with the topic. The goal is to more broadly inform the WTC community about this important topic and its implications for WTC research.

## 2. Data Combining Methods

**Figure 2:**
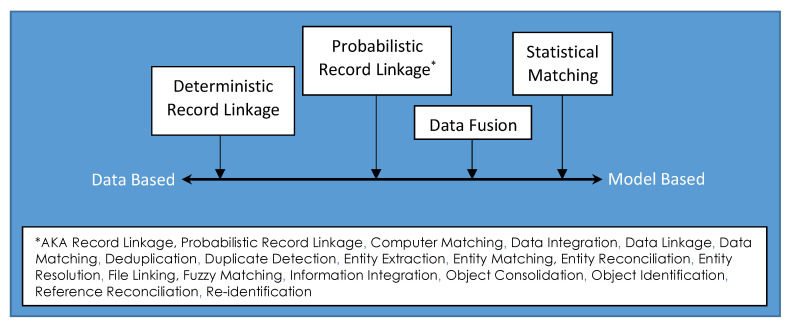
Ranking of data combination techniques from data based (deterministic) to model based (probabilistic).

Several disciplines have contributed to the data linkage literature. Unfortunately, one side effect has been a lack of consistent language surrounding record linkage research efforts. The result has been a variety of terms that are used for the same or similar concepts; Figure 2 lists some of the names indicating the same general process of record linkage. Another point of confusion arises from the fact there are other data combining processes—processes that are not record linkage—with names that are very similar to the ones that do indicate record linkage. To assist the reader, we briefly describe these other data combining processes. 

Probabilistic record linkage is one example of multiple data combination techniques. Figure 2 categorizes the different data combination processes that have been developed over the past 70 years. The oldest and the most data driven of the processes is called deterministic record linkage. This is a rules-based approach, which in its simplest form is simply a data join: Records sharing the identical key are combined into a single data record. In this case, a unique identifier, or a set of identifiers (called a key) that uniquely label an individual, are available for almost every record in one or more lists. If there is no universally valid key that is available, then more complex logic can be used to distinguish links from non-links. A further level of distinction is between unduplication (also called deduplication) and linkage. It is possible to perform the procedure of record linkage within a single list of records; this process is used for lists that contain multiple records all representing the same entity. In the case of two or more lists, both deduplication and linkage can occur.

In our experience, deterministic record linkage is either infeasible or only available for a subset of records for multiple reasons. In the case where a unique identifier is available, errors or missing values might prevent some proportion of records from being linked. More often, no unique identifier is available, and a simple, fixed rule will result in either too many links that are invalid (which is called a Type I error) or true matches that remain unlinked (which is called a Type II error). Probabilistic record linkage starts with data for one or more fields within each list and uses a probability model to determine the likelihood of a pair of records representing the same entity. For example, the name John Smith is a very common name, and therefore the likelihood that two records with the name John Smith really represent the same person is low. Jana Asher, in contrast, is a relatively rare name, so two records with the name Jana Asher are much more likely to represent the same person. Combined with a date of birth, a rare name can almost serve as a unique identifier.

Data fusion, distinct from record linkage, involves the merging of records that represent different entities for the purpose of obtaining a better understanding of the concepts represented by the data. A simple example might help illustrate what data fusion accomplishes. Imagine that two distinct datasets each have spatial data for different non-overlapping coordinates, and some set of covariates that are different for the different datasets, such as temperature in one dataset and elevation in the other dataset. We can combine the datasets and use interpolation to estimate the temperature and elevation for all data points, creating a more complete set of information for the area. The interpolation makes this a less data-driven process than probabilistic record linkage, in that synthetic data are formed from a combination of the existing data and probability models.

Finally, the most model driven of the techniques is called statistical matching. In statistical matching, records with similar characteristics across multiple covariates are matched together to compare values for a different set of variables. One application for statistical matching is to determine a treatment effect in the absence of a control within a randomized experimental design. Since randomization could not or did not happen, the alternative is to find a non-treated entity with characteristics as similar as possible to the treated entity, and pair those records for comparison. Another example is a demographic one; if a researcher wants to compare two variables that are on different datasets when there are not enough appropriate comparison fields for probabilistic record linkage, then statistical matching can be used to link the most appropriate records to each other.

The remainder of this paper will be focused on probabilistic record linkage. Section 3 will briefly introduce the history of probabilistic record linkage. In Section 4, we will discuss the basic Fellegi-Sunter model, machine learning record linkage techniques, and Bayesian modeling for record linkage. Finally, Section 5 and Section 6 will discuss some of the issues that arise in large record linkage projects, such as practical data management considerations and also ethical concerns.

## 3. Historical Context

The origins of record linkage as a field begin at the end of World War II; the original papers on record linkage related to family structure in the United States and elsewhere [1,2,3] and a population registry in Canada [4]. Genealogical researchers also began to explore the idea of linking records from different historical files [5]. Finally, the U.S. government began its decades-long exploration of the idea of linking administrative records and census records to create intercensal estimates [6].

The first mention of computer-assisted record linkage in the literature occurred in 1959, when Harold Newcombe laid the groundwork for probabilistic record linkage in an article in *Science* [7]; around that time, about 4–7 papers on record linkage were published each year. Two years later, a large government record linkage study began in Oxford [8]. In public health, record linkage studies related to radiation exposure and genetic research were beginning; 1963 brought the first linkage project related to psychiatric data [9]. More importantly, specific research on improving record linkage processes began with an article on optimal methods for matching names [10]. The rate of research was increasing—researchers produced 16 papers related to record linkage in 1962, which increased to 40 papers in 1966—but a rigorous framework for probabilistic record linkage did not yet exist.

The breakthrough came from the research of Ivan Fellegi and Alan Sunter between 1967 and 1969 [11]. Over that time period, the Fellegi-Sunter model was formalized, and it has remained the basis for most of the probabilistic record linkage work done since that time. By the time Fellegi and Sunter provided theoretical support for a probabilistic model developed by Newcombe, there were large record linkage centers in Great Britain, Canada, and the United States, and the practice had spread to other countries as well [12,13]. The primary applications were still in public health, demography, and genealogy. By 1970, at least 100 papers were being published on record linkage (deterministic and probabilistic) each year.

From about 1970 to 1999, most of the record linkage research was occurring at national statistics agencies. By the late 1980s, over 300 papers were being published annually, and the first books related to record linkage (both probabilistic and non-probabilistic) were being published. Much of the work had to do with finding optimal string comparators and improving the efficiency and accuracy of record linkage algorithms. In the meantime, computational power continued to grow, allowing larger and larger datasets to be linked in shorter and shorter time periods.

By 2000, the computer science field had discovered record linkage and began to develop machine learning algorithms [14]. Over the next decade, record linkage went from being the province of large governmental organizations that could afford large mainframes to being available to any researcher with a decent desktop computer. By 2010, the first Bayesian record linkage papers were being published. The amount of research was growing at a phenomenal rate; while about 1000 papers related to deterministic and probabilistic record linkage were published in 2001, by 2018 that number had grown five-fold.

The literature about record linkage has literally grown exponentially; a search of Google Scholar yielded over 5000 papers for 2018 alone (see Figure 1). Although Google Scholar hits are not equivalent to individual published papers, it is safe to conclude that there have been thousands of unique papers related to record linkage published per year for the past several years. Over the decades, there have been reformulations of the original problem, development of new theoretical models, and refinements of existing methodologies and software.

## 4. Methodologies

### 4.1. Fellegi-Sunter Model

Fellegi and Sunter’s model is based on a decision rule as to whether a pair of records—these could be two different records from the same list, or a record from one list being compared to a record from another list—are to be linked (imputed to be a match, i.e., the records represent the same entity) or not. It was developed in the context of large governmental statistical agencies, and its original purpose was to link across records representing people. Typically, there are multiple fields that are directly compared for each pair of records under evaluation. For example, in Table 1, we demonstrate a pair of records being compared over five different fields. A decision must be made as to whether each pair of fields agree or not; here, the meaning of agree is flexible. The pair of fields might be identical or close enough by some understood metric; for example, in the case of year-of-birth, no more than one year apart. Because this decision is not a direct part of the Fellegi-Sunter theory, we will ignore these distinctions for now and discuss the agreement decision process later in more depth.

During the linkage process two specific probabilities must be estimated for each pair of fields. The first is the probability that the fields agree given that the underlying entity represented by the records is truly the same (both records being compared represent the same individual), and the second is the probability that they agree given that the underlying entities represented by the records are not the same (i.e., they represent two different people). The former probability is called the **m** probability for the field, and the latter probability is called the **u** probability for the field.

The **m** probabilities for the fields are typically close to, but not exactly, one. When the same entity generates two records (i.e., two records come to be for the same person), there is always a possibility that those records will not match due to human error or other random processes (like optical character recognition error). For example, in Table 1, the first name of the underlying individual is listed as “Jana” and “Jane” in the two records being compared. Jane is a much more common name than Jana, so it is quite possible that Jana includes a typographical error. However, because Jana is a rarer name, it is also possible that a data entry clerk inadvertently created an error by assuming that the name was supposed to be Jane in the first place.

In contrast, the **u** probabilities for the fields vary quite a bit. In Table 1, the fifth field is gender; there is around a 50% probability that two randomly picked records will match on gender; the probability is not exactly 50% due to the sex ratio being not exactly 1:1 and non-binary gender designations. In some record linkage implementations, the first field, first name, will have a different **u** probability depending on the frequency of the name in the population represented by the list(s) being compared. For example, if there are two lists being linked, and each of the lists represents the United States population, then the probability of the name “Jana” is about 0.00015; the probability of the name Jane is about 0.001 [15]. At the time of access, the web site howmanyofme.com claimed that 51,210 out of 330,389,658 individuals in the United States had the first name Jana, and 412,987 individuals in the United States had the first name Jane, making Jane 8 times more likely to have coincided by chance.

Once the determination is made as to whether a field agrees or not, a weight is assigned to it. If the field is determined to agree, the weight is log_2_(**m**/**u**), which is called the agreement weight; if the field is determined to not agree then the weight is log_2_((1 − **m**)/(1 − **u**)), which is called the non-agreement weight and is always negative. The field weights (either agreement or non-agreement) are then summed to create an overall match weight for the pair of records. As can be seen in Table 1, larger **u** probabilities lead to relatively smaller weights, which makes sense: Matching on gender, for example, does not make it much more likely the two records represent the same entity; however, matching on a rare last name does.

In the Felleg-Sunter process, once each pair of records has been assigned a match weight, the pairs are then put in order of their weights, with the largest weights coming first. At that point, two cut-offs can be set to separate the list of pairs into three sections. The top section’s pairs are considered the “links”; that is, the pairs of records that the process has determined (in all likelihood) represent the same person. The bottom section’s pairs are considered the “non-links”; that is, the pairs of records that the process has determined do not represent the same person. The middle section will be reviewed, and a decision will be made individually for each pair as to whether it is a match. Note that the Fellegi-Sunter theory does not provide guidance for setting these thresholds, only that Type I error will be minimized for a fixed Type II error, or Type II error will be minimized for a fixed Type I error. Fellegi-Sunter does suggest that manual review of pairs over a range of weights can be used to determine above which weight pairs are almost certainly matches and below which weight pairs are almost certainly non-matches. More recently, record linkage software packages have included algorithms for determining optimal thresholds based on the record linkage weights.

We have necessarily skimmed over a few details in this process. One is the method by which each pair of fields is compared. For example, names are often pre-processed to standardize spellings and convert nicknames to formal names, after which they are compared either exactly or through a string comparator. When a string comparator is used, it provides a distance measure such as the one developed by Matthew Jaro and William Winkler [16]; in short, the Jaro-Winkler distance creates a function of the length of the name in each record, the number of matching characters in the name across the two records, the number of transpositions (swapped letters) in the name across the two records, and the size of any common prefix at the beginning of the name across the two records. Pairs with shorter Jaro-Winkler distances are more likely to be matches, albeit the process of adjusting weights for distance can be complicated. In the case of numbers, such as year of birth, often a certain degree of difference will be allowable; for example, if the year given in one record is within five years of the year given in the second record.

The other process not yet mentioned is the choosing of which pairs of records to compare. In record linkage processes with large datasets, it is usually computationally infeasible to compare every possible pair of records; for this reason, a blocking strategy is often used. Blocking is the process of restricting the comparisons to only pairs of records that agree on a set of specific fields. For example, if in a given blocking pass, both ZIP code and year of birth are used as the blocking variables, then only pairs of records with the exact same ZIP code and year of birth will be compared. This tremendously reduces the number of pairs of records to be compared; however, if there is an error in a record’s ZIP code or year of birth, a true match might be missed. For that reason, in large record linkage projects, several blocking passes are used. For example, for a first pass, only records with identical ZIP codes and year of birth will be joined into pairs. In a second pass, only records with the same first and last names will be joined into pairs. When multiple blocking passes are used, they can be sequential (only records not linked in the first pass are linked in the second pass), or they can be overlapping (all records are run through all passes), which will require further subsequent unduplication.

### 4.2. Machine Learning

The Fellegi-Sunter method remained the only well-known algorithm for probabilistic record linkage through the end of the 20th century. Advancements in the method had to do with processing speed, the use of expectation-maximization algorithms to determine match weights, creation of string comparators, and applications to new types of records. Eventually, practitioners began to realize that the Fellegi-Sunter algorithm was akin to a type of machine learning called naïve Bayes.

Basically, both the Fellegi-Sunter model and naïve Bayes models rely on the concept of conditional independence; in the case of record linkage, this means that the agreement statuses for comparison variables are independent of each other under the condition of the true match status. However, this is not always the case. An example that readily demonstrates when comparisons are not independent that arises frequently in person-level linkage is for the variables first name and sex. For first name, the probability that a non-matched records pair (i.e., one where the two records making the pair do not represent the same person) will agree (by chance) on first name will be quite low, less than 1%. The probability that a non-matched pair agree on sex is approximately 50%. However, in those cases where the non-matched records by chance agree on first name, the probability that sex agrees is much higher. That is, if we have two different people who have the first name “Mary”, almost certainly they will agree on sex (an important exception is those who are transgender). Because the conditional probability of sex agreement differs from the unconditioned probability, the agreement status for sex is said to be non-independent of the agreement status for first name, and this fact has implications for the probability model used for evaluating the likelihood of matching.

In machine learning, probabilistic record linkage can be replaced by one of several classification algorithms. In some cases, probabilistic record linkage becomes a supervised learning problem; that is, a training set of data is used to “teach” a classification algorithm, such as a decision trees, support vector machines, ensemble methods (such as random forests), or conditional random fields. The classification algorithm used for a specific problem is dependent on the type of data being linked; in most cases, the methodology requires that there be a large number of records in the datasets, as they must contain enough records to allow extraction of a sufficiently large training dataset. Decision trees have been used by business to unduplicate customer lists and build customer profiles that contain purchase histories [17]. When there is a smaller amount of training data available, support vector machines are a feasible alternative approach. Examples of applications for support vector machines include the matching of citations across CiteSeer datasets and unduplication of lists of restaurants [18]. Ensemble methods have been used effectively for information extraction from a large quantity of textual data sources that are continuously changing; for example, during a worldwide web search [19]. Finally, conditional random fields have been used for entity recognition; an example is a web search for computer science concepts and the name of their inventors for the purpose of constructing a table of information such as those found in Wikipedia [20].

Once a classification algorithm is selected, the training dataset is randomly selected from the larger collection of records; often, clerical review is used to determine which pairs of records represent true matches and which do not. Using the training data, the probabilities of a match given a particular agreement pattern across the fields is determined. For example, in Table 1, five fields are compared; the pattern “01101” represents the pattern of matches across the five fields; a zero represents non-agreement for a field, and a one represents (complete) agreement. Once the agreement fields are classified as to their probability of being a match, that information is used for new pairs of records to quickly assign them the appropriate match probability.

An alternative option is an unsupervised method; that is, a method that does not rely on training data. An example is *k*-means clustering. In *k*-means clustering, for each pair, a measure of similarity for each field is calculated. Unlike the match weights in the Fellegi-Sunter algorithm, these measures are set up so that 0 indicates a perfect match for the field, and higher numbers indicate the fields are less similar. A ceiling value of 1 can be set to mean that the fields completely disagree.

Multiple similarity measures have been used within unsupervised record linkage. For numeric data, similarity is fairly easy to measure. For text-based fields, the similarity measures are more complex. The Damerau-Levenstein distance, developed in the 1960s, is a single numeric measurement based on the comparison of two text strings for four common transcription errors: insertions, deletions, substitutions, and transpositions [21,22]. The Jaro-Winkler distance, described earlier, can also be used as the basis of a similarity measure [16]. In the case of the Damerau-Levenstein distance, a similarity measure can be found by taking one divided by the calculated distance; in the case of the Jaro-Winkler distance, the similarity measure is found by taking one minus the calculated distance.

For some record linkage tasks, semantic matching—that is, matching across the meaning of two strings of text, or determining if two strings of text represent related concepts—is the desired outcome. One class of similarity measures for semantic matching requires a known set of linked concepts against which the two strings of text can be compared; the similarity measure is based on how “close” the two strings are in the map of known concepts. A different type of similarity measure is based on a large set of text documents in which the concurrence of the two strings of text is flagged; the similarity measure is then based on how frequently the two strings of text are found together. However, recent work has suggested that semantic matching is of highest quality when multiple similarity measures are aggregated to create a single measurement system [23].

Note that when similarity measures are used, a binary decision for each field is no longer required. For example, in Table 1, the names “Jana” and “Jane” might be assigned a value based on their Jaro-Winkler distance; because they are very similar, this number will be very close to zero. Overall, a vector of five numbers would represent the pair of records in Table 1, with each element of the vector representing the agreement value for one of the five fields. First name would have a value close to zero, last name and date of birth would each have a value of zero, address would have a value close to one, and gender would have a value of zero.

Once each of the pairs of records has an associated similarity measure vector, the distance between different pairs of records is measured. One common distance measurement is the Euclidean distance; this is determined by summing the squared distance—that is, the difference in the field value between two record pairs—across all the fields being compared. Record pairs that are “close” to each other according to the distance measure are formed into clusters. Other possible distance measurements include the Manhattan distance, which replaces squaring the difference in field value between two record pairs with taking the absolute value of that difference, and the Hamming distance, which is used when similarity measure vectors contain only binary (0,1) values. In record linkage, typically three clusters are formed to match the three regions of match weights in the Fellegi-Sunter algorithm; one cluster represents the links, one the non-links, and one the possible links.

### 4.3. Bayesian Record Linkage Techniques

Bayesian record linkage techniques are the most complex that have been developed to date and should be considered experimental. Bayesian techniques rely on probabilities of a match or non-match for specific agreement patterns that are either based on expert opinion or on previous projects. These prior probabilities are then combined with a new record linkage process involving new lists. At the end of the process, a posterior probability of match or non-match is determined for the record pairs, which allows the determination of links and non-links. Like machine learning processes, Bayesian record linkage can incorporate non-binary decisions as to whether a field agrees or disagrees for a given pair of records.

Early efforts in Bayesian record linkage involved expansions of the Fellegi-Sunter algorithm. The **m** and **u** probabilities were assumed to follow a probability distribution of some type; for example, a uniform distribution with all possible probability values being equally likely, or a Beta distribution, with the parameters of the distribution set based on expert knowledge. The optimal (mean) values from the posterior distributions of the **m** and **u** probabilities were then used to create the match weights and complete the linkage process.

Later work in Bayesian record linkage expanded the machine learning algorithms discussed earlier. Modifications included the use of training data to set the prior values for **m** and **u,** and the use of the Dirichlet probability distribution for the prior distributions of **m** and **u**, which allowed the modeling of conditional dependence between those probabilities [24]. More recent work has embraced the classification algorithms of machine learning. For example, a Bayesian model can be created that includes parameters that define partitions in the data, or groupings that are most similar, and parameters that define the probabilities for the values that the similarity measures can take. For example, if five fields will be compared during the linkage process, and the similarity measure for each field takes three possible values, then there will be 15 similarity measure parameters needed, one for each level for each field. Prior distributions are determined for the partition parameters and the similarity measure parameters, and the posterior distribution of all these probabilities together allows the clustering [25].

Although multiple record linkage models have been developed using Bayesian methods, in our experience, there is no software package available that allows a non-expert user—that is, a person that has not learned Bayesian statistics and record linkage at a level expected of a person who has a Masters or PhD in statistics—to complete Bayesian record linkage. Thus, for this type of procedure to be run successfully requires a statistician or data scientist who is trained in Bayesian computational methods. Further, it is our experience that while Bayesian record linkage seems promising, it is not so well developed as to warrant its use over the more familiar Fellegi–Sunter-based processes.

### 4.4. Open Research Questions

Record linkage research is occurring at phenomenal speed. A driver in the explosion of research around record linkage is the growth in the count and size of available datasets. Many recent advances in machine learning algorithms for record linkage have been motivated by the demand for more efficient and accurate web search tools. A related area is the need to be able to match across different languages as more countries are developing their own large data repositories. A final important area of record linkage research is based on the advent of repositories of non-traditional forms of data—i.e., sound clips, visual representations, and multi-dimensional surfaces. Matching these types of data effectively and efficiently will require more sophisticated record linkage methodologies than are available today.

A separate set of research questions is more practical in nature. In some contexts, researchers are being asked to obtain informed consent from all persons whose records will be matched for research purposes. In other contexts, researchers cannot access the large datasets that can be used for social and health-related research due to concerns around privacy and confidentiality. Current research topics related to these concerns revolve around privacy-preserving record linkage and understanding the bias introduced by the requirement for informed consent [26,27]. We turn to further discussion of the practical considerations around record linkage next. 

## 5. Practical Considerations

Theoretical models are the basis of record linkage methods; however, it is the practical considerations of record linkage that require the most effort to understand and problem solve. We will now review these practical considerations and make suggestions as to best practices.

### 5.1. Data Cleaning and Standardization

Due to the variety of ways in which records of human beings are transcribed, performing standardization prior to matching is almost always required. Organizations that frequently match across large datasets have standardization processes in place; for example, the U.S. Census Bureau will process data to standardize the sex field (e.g., changing “f”, “Female”, “female”, and various misspelled versions of “female” all to “F”) and the format for date of birth, remove honorifics (e.g., “Mr.”, “Mrs.”, “Dr.”) and convert nicknames to formal names, and conduct address geocoding. However, 9/11 exposure and health data have been and continue to be collected by a variety of research teams both for research and clinical purposes, making data standardization a challenge. Commercial packages exist that will automate some of these processes. These packages range in prices and capabilities, and new tools are constantly being developed. For a recent example, please see smartystreets.com/pricing [28].

If multiple organizations plan on sharing data, a common format for data entry is a time saving but not complete solution. Organizations can and do plan to use a common format for sex, date of birth, and honorifics; however, multiple human processes will continue to cause a certain level of error in the fields collected. For example, “age heaping” is a known problem in the demographic field; individuals have some probability of reporting their age to the nearest “5” or “0” instead of exactly (e.g., a 49-year-old might report being 50). Individuals also vary in how they report information about themselves such as name; nicknames might be used in place of first names, and middle names may or may not be included.

### 5.2. Missing Data

Missing data is a problem within any record linkage technique and can be due to a variety of factors. For example, participants may neglect to answer a question due to the sensitive nature of some topics, such as post-traumatic stress disorder symptoms. In addition, because most 9/11 research is longitudinal in nature, attrition has become a more salient problem over time. Even clinical data may be incomplete if a patient misses multiple follow-up visits or leaves a provider altogether.

Incomplete data have been handled in several different ways. Some practitioners simply remove any records with missing data; however, this can cause quite a bit of bias in any estimation done with the linked data [29]. Other practitioners set the field weight to zero if one of the fields is missing, because from a probability perspective, the comparison is identical to a comparison that did not include that field.

Some research has been done on alternative techniques for handling missing data. One possibility is to re-distribute the value of the match weight for the field containing the missing data to all other fields that are found to match, and the value of the non-match weight for the field containing the missing data to all other fields that are found to not match. In that way, the pair of records has similar “weight” to other records without the missing data. Another possibility is to impute the match status of the field containing a missing value based on the match status of the other fields being compared. Ong et al. [30] found that both methods outperformed setting the weight for the field with missing data to zero.

Finally, direct imputation of the missing values through a regression model or other modeling technique is a possibility, although it adds additional error into an already complex process [29].

### 5.3. Error Measurement

Measurement of error in record linkage processes is essential for providing an accurate measure of variance during estimation using linked data. The most common techniques for determining error rates include the use of a gold standard field (a unique identifier that is available for only a subset of records, precluding its use for deterministic record linkage), a clerical review process (which often is too costly and time consuming to consider), and model-based approaches. Resnick and Asher [31] propose a new Type I and Type II error measurement technique that requires further exploration to determine which record linkage conditions are required for its use. Finally, simulation techniques, where the exact match status is known, can be used to determine approximate error rates in specific record linkage contexts.

### 5.4. Software

Given the multiple potential record linkage techniques available, software for record linkage is not difficult to find. However, all benefit from a decent knowledge of record linkage techniques and the underlying statistical models to use. Recently Karr et al. [32] reviewed multiple software options for record linkage: R (Version 3.4.0, RecordLinkage package; no longer supported), Merge ToolBox (MTB, Version 0.75), Curtin University Probabilistic Linkage Engine (CUPLE), and Link Plus (LP, Version 2.0). They found very few differences in the performance of the packages, despite each relying on different matching algorithms. Other software includes Match*Pro, a relatively new linkage software developed by information technology company IMS, Inc that is currently being utilized by several cancer registries and also an R package (fastLink) using methods developed by Enamorado et al. [33]

Typically, individuals with the data editing expertise to appropriately perform record linkage using the available free or low-cost software packages will be able to import data that starts in a variety of formats. In most cases, data stored in a commercial database can be exported into a comma delimited file (.csv); conversely, most software packages will be able to import comma delimited data.

### 5.5. Data Sharing

If different organizations are sharing data files, a basic set of rules should be determined on how files will be transferred. Typically, the areas to consider are:

1. Data format. If different organizations use different data management systems, direct transmission of files without conversion into a commonly accepted format will cause issues. As mentioned before, most statistical and/or record linkage software can accept a comma delimited file as input.
2. Data description. A separate file describing the data should be included; information about a dataset is typically called the “metadata”. Metadata includes information on how the data were collected, a definition and value range for each variable in the dataset, and any restrictions on the data usage.
3. Confidentiality agreement. All employees that will have access to the shared data should agree, preferably through signing a contract with the organization providing the data, to maintain the confidentiality of the data.
4. Length of time data are available. If the data are only being shared for a limited time period, the parameters for that time period should be outlined prior to data transmission. In addition, requirements for the “disposal” of the data once the time period is over should be agreed upon in advance.
5. Institutional Review Board (IRB) requirements. In some cases, IRBs have determined that record linkage does not fall under human subjects protections; in other cases, IRBs have regulated record linkage projects. Any IRB restrictions that are already in place regarding the data to be shared must be understood by all parties engaging in the record linkage process.
6. Health Insurance Portability and Accountability Act (HIPAA) requirements. In some cases, IRBs have required that informed consent be obtained from the individuals whose data will be used in the record linkage process. If data are being transferred between organizations, a new informed consent agreement might be required.
7. Secure transmission of data. The protocol for data transmission should be agreed upon in advance and should have appropriate security protocols. Record linkage projects sometimes have stringent security requirements. For example, sensitive de-identified datasets sometimes require researchers to use a computer within a secured data center, separate from the Internet.

### 5.6. Documentation of Record Linkage Processes

Because record linkage is often performed by a limited set of individuals with the appropriate expertise, documentation of the linkage process is an essential part of the record linkage methodology. In other words, organizations should develop a pre-determined structure for a required report to be completed for every record linkage project. Such a report might include the following:

1. The names of the individuals that complete the record linkage project.
2. The purpose of the record linkage project; for what analysis will the linked data be used?
3. The precedents of the record linkage project, if part of a longitudinal study, and where the documentation for previous iterations of the project can be found. Please note that even when record linkages are to be repeated at different time points with new data, each linkage should still have its own documentation.
4. The metadata for each file to be linked, including:
  - The names, file positions, and descriptions of the variables;
  - The data collection process for the dataset;
  - The organizational source (in house or outside organization) for the dataset;
  - The date of acquisition of the dataset;
  - The contact information for the person from which the dataset was obtained; and
  - Any rules regarding the use and disposal of the dataset.
5. The software that is used for the linkage.
6. The methodology for the linkage, including:
  - How many passes are performed when linking the data;
  - If blocking is used, which variables are used as blocking factors during which passes;
  - If parameters are set ahead of the process—for example, if prior values for m and u probabilities are required—what values are used for each pass.
  - Any information as to how linked pairs, non-linked pairs and possible linked pairs are determined. For a standard Fellegi-Sunter process, this information includes the range of match weights for each of these groups; for a machine learning unsupervised clustering process, this information includes the mean value for each of these groups and the range of distances that determine which pairs are clustered into which groups.
  - Some measurements of the error rate in the linkage process; ideally, these include a false-positive and false-negative rate.
  - A definition for any new variables created during the linkage process.
7. Any known limitations of the record linkage process, including issues with the data that might have made record linkage problematic, known methodological issues related to the algorithm used for the linkage, and specific issues that might have arisen during the record linkage project.
8. The final disposition of the linked datasets, if they are not available indefinitely.
9. In some cases, the linked data will be stripped of all identifiers, allowing the resulting dataset to be freely used without confidentiality constraints. If this is the case, the process of removal of identifying information should be outlined.

## 6. Ethical Considerations

### 6.1. Privacy Preserving Record Linkage

Privacy and confidentiality are important considerations for all health data, particularly those surrounding stigmatized mental health conditions. For these reasons, data sharing is challenging between 9/11 cohorts. One option is a collection of techniques called privacy-preserving record linkage (PPRL) [34]. First, review and approval/exemption must be obtained from IRBs or other oversight committees that understand the protections PPRL affords. Then the organizations in question pre-process the data to standardize the format for each field to be used in the linkage process. The next step in PPRL is some type of encoding of private information using a secure coding scheme. For example, phonetic encoding creates similar codes for strings that have similar pronunciations; however, when done correctly, the encoded strings look nothing like the original strings. To ensure no one party can “back-translate” the encoded strings, an algorithm can be used that requires separate inputs from each party; without all the inputs, the encoding algorithm cannot be reversed. Once the encoding takes place, there are at least two linkage approaches: (1) The two parties interested in the linkage each share their file with the other, allowing the match to occur, or; (2) both parties share the data with a third party, and the third party performs the match and returns the linked pairs.

There are multiple existing organizations that can serve as a third party during a linkage project. Data61, a digital research network based in Australia, developed a set of digital tools for PPRL called Anonlink [35]. Data61′s Anonlink was composed of open source software packages, and Data61 was the “third party” for Anonlink’s PPRL. Similarly, Canada’s PolicyWise for Children & Families, a non-governmental research organization, has developed the LinkWise package for PPRL [36]. In the case of LinkWise, there is a fee for usage, and PolicyWise for Children & Families serves as the “third party” match service.

It is possible to complete PPRL without paying a third party. There is a freely available PPRL package in the statistical software platform R [37]; however, a significant understanding of statistical programming is required for usage.

### 6.2. Biases in the Record Linkage Process

There are multiple potential sources of bias in record linkage. Differential record linkage refers to the fact that data of lower quality typically result in less probability of linking data representing the same entity. If lower quality data were randomly distributed among the records being matched, this might not be a contentious issue; unfortunately, through studies like the ones completed by Lariscy [38,39], we know that underrepresented groups are more likely to have data of lower quality, and therefore are more likely to have higher levels of error in the linked data associated with them. Researchers intending to use data files compiled through record linkage need to be aware of the biases that could affect their findings due to differential record linkage; for example, this would be an issue for linkages of persons in New York City, considering its ethnic and racial diversity.

Another source of bias is introduced through pre-processing protocols and data standardization systems for record linkage; the majority of these have been developed in an English-centric linguistic cultural context. For this reason, they are not as useful in other language contexts. In their 2016 paper, del Pilar and Bailón address this issue by creating phonetic encoding algorithms specific to Spanish names to allow lower quality Spanish name data to be matched [40]. Similarly, Munkhjargal et al. [41] develop specific algorithms for Mongolian names, and Ma et al. [42] discuss data linkage in the Asian context.

Gender also plays a role. Data linkage often assumes gender is fixed but that is not the case for those who are transgender or non-binary. In addition, algorithms that match on last name put women at greater risk of not successfully matching because they often change their surnames at marriage.

Record linkage processes that occur in multicultural contexts might encounter biases due to name standardization algorithms that are not appropriate for many of the naming conventions of the different subpopulations represented in the data. Practitioners must develop tools that account for naming conventions of those subpopulations; rules based on Hispanic and Asian naming conventions are the most likely to be needed.

## 7. Conclusions

We have addressed multiple topics related to record linkage, including the three types of underlying models for the probabilistic aspects of record linkage, the basic methodologies by which these models are employed, practical considerations such as data standardization and data sharing issues, and potential sources of bias such as differential rates of linkage across minority populations.

The success of a record linkage project, however, is dependent on the organizational structures that allow it to continue. In large data-driven organizations such as the U.S. Census Bureau, data governance structures are formalized through understood protocols for data sharing and a specific review committee for record linkage requests. Specific funding towards a team of record linkage experts is an annual line item in the organizational budget and may be a consideration as 9/11 research needs evolve.

Record linkage cannot be accomplished without the appropriate in-house expertise. Expertise in information technology is not adequate; at minimum, an organization must have access to a data scientist or statistician that understands the probability models on which record linkage methods are built and can use statistical software packages to complete record linkage tasks. This individual must have enough technical expertise to read and understand the scientific literature related to record linkage as new methods are developed. Finally, they must be able to adapt the techniques being employed to complete the record linkage as data with different levels of quality are included in the process.

In short, while record linkage can create avenues of research that might not otherwise be available, it is not an easy process to complete. Even the most expensive record linkage software requires a skilled practitioner and detail-oriented documentation of the steps taken for a specific record linkage project. Without institutional commitment to providing adequate resources and informed oversight for record linkage efforts, attempts to link data are likely to end in an unsatisfactory result.

## Figures and Tables

**Table 1:** Pair of records compared during a probabilistic record linkage process. In this case, overall match weight, based on three matched fields and two non-matched fields, is 17.20.

Field 1: First Name	Field 2: Last Name	Field 3: Date of Birth	Field 4: Address	Field 5: Gender
Jana	Asher	10/17/1970	603 Brook Court	F
Jane	Asher	10/17/1970	1111 Jackson Ave	F
**m**_1_ = 0.95	**m**_2_ = 0.99	**m**_3_ = 0.97	**m**_4_ = 0.95	**m**_5_ = 0.99
**u**_1_ = 0.001	**u**_2_ = 0.00004	**u**_3_ = 0.001	**u**_4_ = 0.01	**u**_5_ = 0.48
log_2_((1 − **m**_1_)/(1 − **u**_1_)) = −4.32	log_2_(**m**_2_/**u**_2_) = 14.60	log_2_(**m**_3_/**u**_3_) = 9.92	log_2_((1 − **m**_4_)/(1 − **u**_4_)) = −4.31	log_2_(**m**_5_/**u**_5_) = 1.31

**m** is the probability the fields agree given they represent the same entity; **u** is the probability the fields agree given they do not represent the same entity.
